# An Analysis of Preliminary and Post-Discussion Priority Scores for Grant Applications Peer Reviewed by the Center for Scientific Review at the NIH

**DOI:** 10.1371/journal.pone.0013526

**Published:** 2010-11-17

**Authors:** Michael R. Martin, Andrea Kopstein, Joy M. Janice

**Affiliations:** 1 Office of the Director, Center for Scientific Review, Bethesda, Maryland, United States of America; 2 Substance Abuse and Mental Health Services Administration (SAMHSA), Director of the Office of Program Analysis, Bethesda, Maryland, United States of America; 3 Tunnell Government Services, Bethesda, Maryland, United States of America; University of Exeter, United Kingdom

## Abstract

There has been the impression amongst many observers that discussion of a grant application has little practical impact on the final priority scores. Rather the final score is largely dictated by the range of preliminary scores given by the assigned reviewers. The implication is that the preliminary and final scores are the same and the discussion has little impact. The purpose of this examination of the peer review process at the National Institutes of Health is to describe the relationship between preliminary priority scores of the assigned reviewers and the final priority score given by the scientific review group. This study also describes the practical importance of any differences in priority scores. Priority scores for a sample of standard (R01) research grant applications were used in this assessment. The results indicate that the preliminary meeting evaluation is positively correlated with the final meeting outcome but that they are on average significantly different. The results demonstrate that discussion at the meeting has an important practical impact on over 13% of the applications.

## Introduction

Peer Review at the National Institutes of Health (NIH), a United States Government agency that supports biomedical research, has two separate stages: the initial scientific merit review of the proposed research by a panel of peers; and a second level review by the Advisory Councils and Boards for funding Institutes and Centers (ICs). The IC Councils and Boards are composed of scientists from the extramural research community and public representatives [1]. The IC Councils and Boards evaluate the relevance of the proposed science to the mission of the IC, the potential impact of the research, and the IC's concurrence with the initial review. The NIH dual peer review system is mandated by federal statute [2].

The Center for Scientific Review (CSR) manages the review of approximately 70% of all applications submitted to NIH in the first phase of the peer review process. Most of these are standard research grant applications (R01s). The initial scientific merit review conducted by CSR is further divided into two serial segments. The first segment is the assignment of the application to three or more panel members to prepare the critique and provide a preliminary or pre-meeting score. The second stage is for the full panel to review the application and discuss the critiques before they vote a final priority score for the application. This phase is usually in the form of a face-to-face meeting

Grant application peer review has been compared to an art form, combining individual assessments of quality and impact. There are difficulties in quantifying or validating the final results [3] and the rating scale has also been challenging [4]. Over the past 20 years the impression has emerged that discussion of applications may have little impact on final meeting scores as reflected by the change in the average of the independently derived pre-meeting scores and the final score. While many studies on the CSR peer review process have been published [e.g. 5], [ 6], [ 7], [ 8], one aspect not examined is the effect of discussion on final peer review outcomes, a focus of this study. In addition, this study will consider the practical consequences of any final priority score change from the average preliminary score.

The data are broken down by: (A) preliminary individual and average priority scores: (B) magnitude of differences; (C), the degree to which the final priority score was outside the minimum and maximum of the individual assigned reviewer's preliminary priority scores; (D), the magnitude of differences as a function of final priority score and (E) the practical consequences.

## Methods

Data were derived from the IMPAC2 Data File maintained by NIH's Office of Extramural Research. The data set included R01 (standard research grant) applications reviewed by CSR for the January 2009 review round. The dataset was a sample of all R01 application reviews.

A research application is defined as clinical when the principal investigator indicated involvement of human subjects in the proposed research by checking “yes” on page 1 of the grant application form in response to a query about involvement of human subjects. Excluded from this definition are applications that identified human subjects in the research and claimed Exemption 4. Exemption 4 applies to research involving the collection or study of existing data, documents, records, pathological specimens, or diagnostic specimens, if these sources are publicly available or if the information is recorded by the investigator in a manner that subjects cannot be identified, directly or through identifiers linked to the subjects. This definition of clinical research captures research on mechanisms of disease, therapeutic intervention, clinical trials, development of technologies, epidemiological and behavioral studies and outcomes as well as health services research.

Study sections that review R01 applications are of two main types: Standard Review Groups (SRGs) and Special Emphasis Panels (SEPs). SRGs are panels that have defined charters describing their areas of scientific expertise. They typically meet three times a year and are comprised of appointed members who serve for four years combined with temporary members who serve once to provide supplemental expertise. The initial peer review panels meet face-to-face and usually include 20 to 35 reviewers.

This analysis includes only SRG panel data. It does not include reviews occurring in SEPs since these reviews use many formats (e.g., face-to face and web-based discussion) that could complicate the interpretation of results. The small number of R01 applications reviewed by SEPs also limits the ability to analyze results from these meetings.

For each application a minimum of three review panel members are assigned to review the application: at least two provide written critiques and one may be only a discussant who adds to comments made by the first two assigned reviewers. All three assigned reviewers provide independently derived “preliminary” priority scores using a standardized set of evaluation criteria.

At the beginning of the panel discussion the assigned reviewers verbally re-state their independent assessments of the appropriate priority scores for the panel. After the discussion the assigned reviewers were asked to verbally re-state their priority scores. These scores may be the same as or different than their pre-discussion preliminary scores. These scores, which are given after the discussion, are not captured and thus were not part of this assessment. These scores would also have been modified by the discussion and not independent.

If an application was deemed to be “non-competitive” (in the lower half, quantitatively of applications reviewed by the panel), by unanimous agreement of the members, it was not discussed at the meeting and did not receive a final priority score [9]. Because these applications were not discussed they did not include a final priority score, and could not be included in this study. Approximately 50% of the applications go on to further discussion by the full panel.

The panels used incremental units of 0.1 in scoring applications from 1.0 (highest merit) to 5.0 (lowest merit). The individual panel members, including the three assigned reviewers, independently and privately score the application after the discussion. The purpose was to preserve confidentially and independence of personal voting. CSR review panel members were allowed to score applications up to 0.5 points outside the range stated by the assigned reviewers' without indicating to the other members of the panel they were ‘out of range’. Reviewers were not allowed to vote outside more than 0.5 outside this range unless they made a statement to the panel since the score could be due to a substantive difference of opinion or fact that had not been fully explored during the discussion.

For the purposes of this study the preliminary score refers to the average of the three assigned reviewers' independent scores, expressed to two decimal places. The final priority score of the SRG, also reported to two decimal places, is the average score of all the voting members of the panel (some members may be in conflict and would not participate in the discussion or vote).

The ICs use the priority scores and a calculated percentile ranking in assisting in their decisions regarding funding [10]. Using percentile ranking enables ICs to integrate the outcomes of multiple SRGs. After the SRG meeting each application that received a final priority score was also assigned a percentile value. The percentile for an R01 application is its relative rank within that SRG. The calculated percentile value for a given R01 application specifies the percent of applications with scores equal to or better than that application, The base used for calculating the SRG percentile for an application is defined by all R01 applications assigned for review by the SRG over three review rounds, whether the application was discussed and scored or not. Because preliminary scores are based on only thee members of the study section there is no base and thus they are not percentiled. Therefore a direct comparison of the preliminary evaluation with the final percentile for the panel is not possible. Thus, comparisons and analysis were limited to priority scores.

## Results

### Description of Sample

CSR conducted R01 reviews in 172 SRGs for the January 2009 Council review meeting dates. Of these, 61 SRGs were included in this sample. The total number of R01 applications reviewed in all SRGs for the January 2009 Council round was 7,503; approximately 50% of these were not discussed and thus did not receive a final priority score [8]. Applications without a preliminary or final priority score were removed from the analysis. Applications with average preliminary and final priority scores from 1.00 to 3.00 were included. After removing unscored applications, there were 1,395 scored applications reviewed in the 61 SRGs, or 42% of the total of all SRG-reviewed and scored R01 applications. SRGs that participated in this study were randomly selected and represent the range of science reviewed by CSR.

### Average Preliminary versus Final Priority Scores

**Figure 1:**
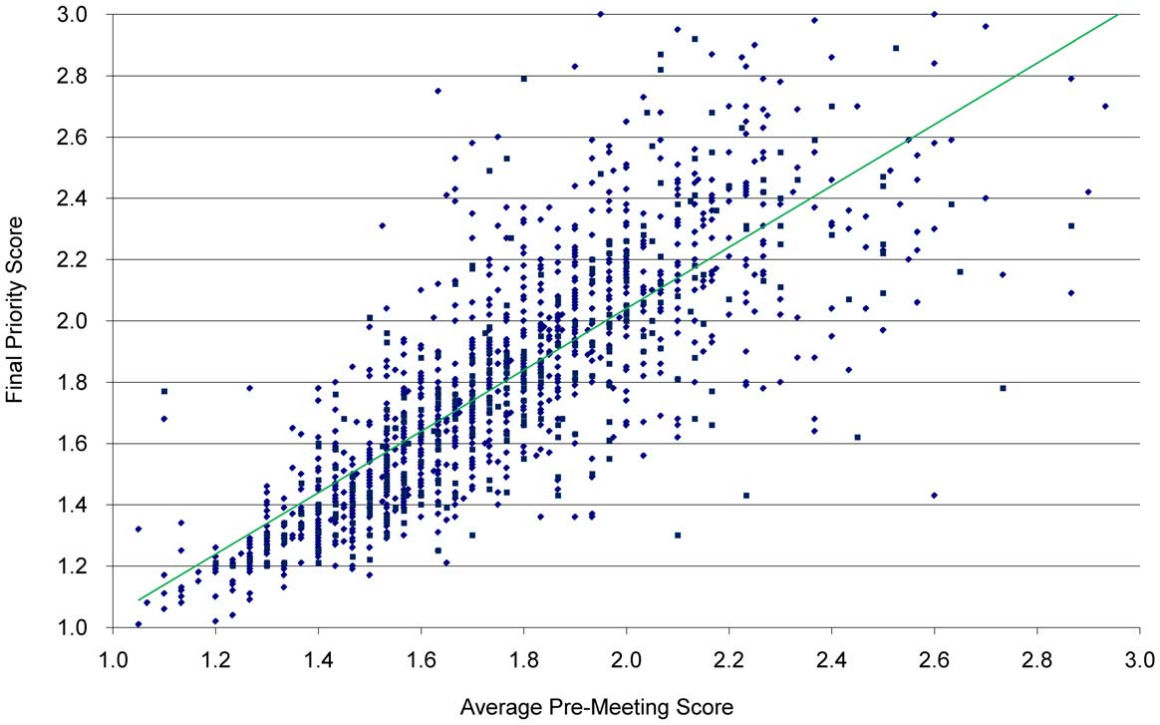
Average Preliminary Score versus SRG Final Priority Score. Preliminary Scores represent the average of the independent R01 priority scores given by the three assigned reviewers; the final priority score is the average of all the scores given by the voting members of the panel. Each data point represents the outcome for one R01 application. The difference between preliminary and final priority scores represents the change between the two values. Applications with differences displayed on the left declined after discussion; those on the right improved.

Figure 1 illustrates the differences between preliminary and final priority scores for all R01s in the sample that received a final priority score of 3.00 or better. Examples of extreme movement include an application with a preliminary score of 2.60 and a final priority score of 1.43 and another with a preliminary score of 1.70 and a final priority score of 2.90. Most differences between average preliminary and final priority scores were in a more narrow range. Overall, the Pearson correlation coefficient is 0.78 (N = 1,395).

### Differences Between Preliminary and Final Priority Scores

Table 1 shows the results of paired-sample t-tests that were used to assess whether the means of the preliminary and final scores were statistically different from each other. Overall, the analyses show that there were statistically significant differences between preliminary scores and final scores for all types of R01s at the p<0.01 level, except for New Investigators. New Investigators represented the only group where the mean difference between the preliminary (1.81) and final priority scores (1.82) were not statistically different.

**Table 1:** Paired T-Test Comparing Average Preliminary and Final Priority Score.

Application Subgroup	N	Preliminary Score Mean ± SD	Final ScoreMean ± SD	Score Difference	t-test	P-Value
New Investigator	295	1.81±0.32	1.82±0.27	0.03	−0.33	0.37
Experienced Investigator	1,110	1.75±0.32	1.79±0.04	0.04	−6	<0.01
Clinical Application	422	1.77±0.33	1.83±0.25	0.06	−4.49	<0.01
Non-Clinical Application	973	1.75±0.32	1.78±0.24	0.03	−3.55	<0.01
Type 1 Application	865	1.79±0.33	1.83±0.25	0.04	−4.15	<0.01
Type 2 Application	527	1.71±0.30	1.74±0.22	0.03	−3.39	<0.01
Overall	1,395	1.76±0.32	1.80±0.24	0.03	−5.26	<0.01

### Magnitude of Differences

**Figure 2:**
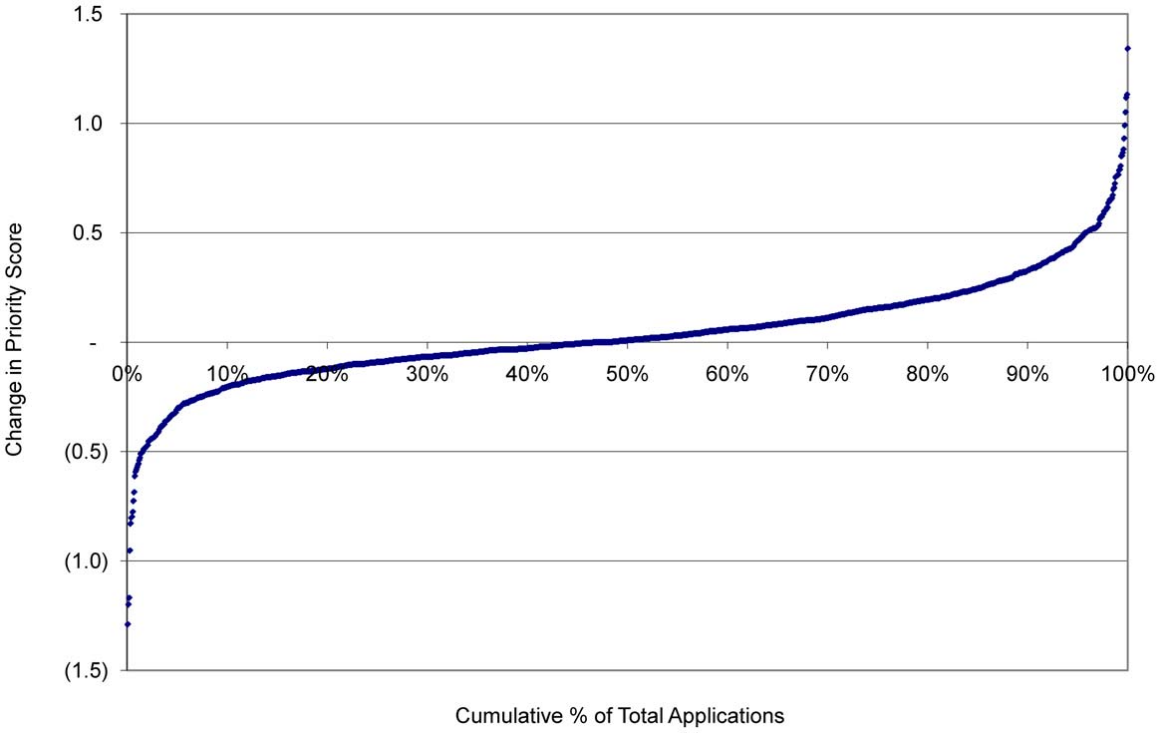
Effects of SRG Meeting Discussion on Final Priority Score. Applications were rank ordered by the difference between the average preliminary and final priority score of individual R01 applications and then assigned a cumulative percent value. Applications with negative values are displayed on the left and improved after discussion; those on the right were worse after discussion.

When the difference between preliminary and final priority scores are rank ordered (Figure 2), only 4% of SRG R01 applications had no change (0.00) in priority score, 45% improved and 51% were worse after discussion. The maximum observed changes in average preliminary score to final priority score was an improvement of +1.29 and decline of −1.34.

### Range of Reviewer's Preliminary versus Final Priority Scores

**Figure 3:**
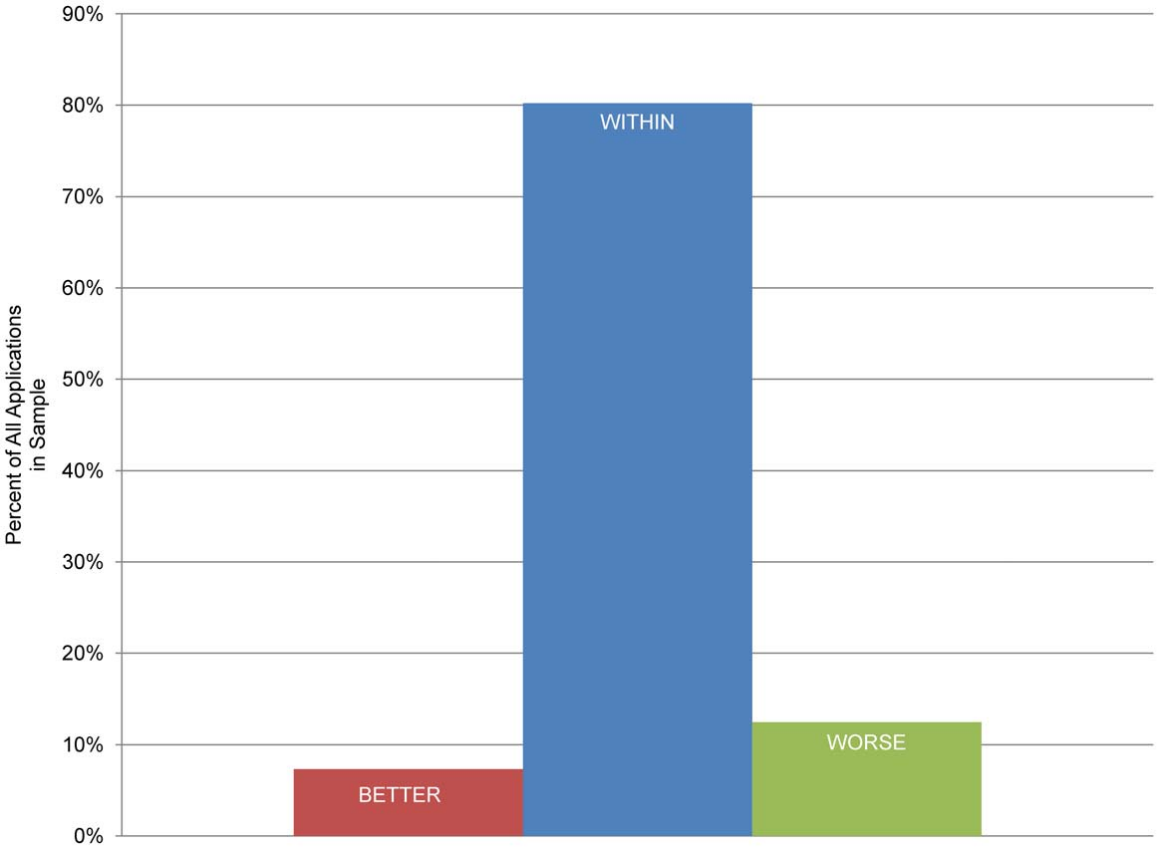
Range of Reviewer's Preliminary versus SRGs Final Priority Scores. The average preliminary priority scores of reviewers may all be the same or represent a range. The final priority score was BETTER, WITHIN or WORSE than this range.

The assigned reviewer's scores established the preliminary score range (e.g., if preliminary scores were 1.2, 1.3 and 1.6, the preliminary score range would be 1.2 to 1.6 – with 1.2 being the best and 1.6 being the worst preliminary score). In aggregate, after discussion, the final priority scores remained within the preliminary score range 80.2% of the time (Figure 3). The balance, 19.8%, were outside of the preliminary range (either better or worse). They were better (lower priority score) 7.3% of the time and worse (higher priority score) 12.5% of the time. Thus, discussion more often increases than decreases the priority score (e.g. preliminary score range 1.4 to 1.6 and final score 1.7.

### Final Priority Score Percentile “Range Band” and Magnitude of Change from Preliminary Score

While there is a significant difference between average preliminary and final priority scores over all, this does mean that there was a practical significance. The importance of substantial changes in priority score, where the preliminary and final priority scores are well beyond any IC funding range, are of lower interest when it comes to issues such as funding. However, there is the potential for high practical impact when either the preliminary or final priority score is between 1.00 and 1.75. In order to better understand the practical consequences of the changes in priority score, applications were sorted into extrapolated percentile ranges or bands that correspond to estimated percentiles as follows:

1. 1.45: approximated the 12^th^ percentile,
2. 1.60: approximated the 19^th^ percentile,
3. 1.75: approximated the 25^th^ percentile, and
4. 1.76 and above: approximated 26^th^ percentile and worse

The base for the percentile can be against all the R01s in either the study section where the application was reviewed (SRG Base) or against all the R01s reviewed in CSR (CSR ALL base). To assign a given priority score to a specific percentile, the “CSR ALL” percentile base was used to estimate these values.

Only 22.6% of R01 applications (Table 2) in the sample had no or minor movement after discussion (defined as 0.05 or less. Also, 42.6% had changed by 0.15 or more (fourth row). Most of the applications (29.3%) that had large changes had final priority score values of 1.75 or worse. Even large changes in this higher range are unlikely to have a practical impact. As an example of a potentially important impact on the final percentile score, an application with an estimated 19^th^ percentile value (160 priority score) before the discussion could have had an estimated final percentile of 12^th^ percentile (145 priority score) after discussion.

**Table 2:** Magnitude of Change from Preliminary Priority Score to Final Priority Score by Defined Ranges.

	Priority Score Ranges				
Magnitude of Change	1.00–1.45	1.46–1.60	1.61–1.75	1.76 to 3.00	Total
less than .05	6.6%	4.9%	3.7%	7.3%	22.6%
,05 to .099	5.7%	3.1%	3.7%	7.7%	20.2%
.1 to .149	3.9%	2.2%	2.7%	5.9%	14.6%
.15 or more	6.7%	3.2%	3.4%	29.3%	42.6%
Total	22.9%	13.3%	13.5%	50.3%	100.0%

### Comparison of Preliminary Score Band with Final Priority Score

The degree and direction of change from the average preliminary scores to the final priority scores included some that got better scores, some that were worse and others that did not change. Analysis of the magnitude of change from one preliminary priority score to a final priority score band (columns, Table 3) provides an indication of the direction and magnitude of change. Cells in Table 3 noted with a superscript ‘A’, show the percent of applications with preliminary and final scores that remained in the same priority score band. Cells noted with a superscript ‘B’ show the percent of applications with final scores that improved vs. the preliminary scores. And cells noted with a superscript ‘C’ show the percent of applications with worse final priority scores than preliminary scores.

**Table 3:** Change between Average Preliminary Score and Final Priority Score.

	Final Priority Score			
Pre-Meeting Average Score	1.00–1.45	1.46–1.60	1.61–1.75	1.76–3.00
1.00–1.45	85% ^A^	11% ^c^	3% ^C^	2% ^C^
1.46–1.60	34% ^B^	37% ^A^	16% ^C^	13% ^C^
1.61–1.75	9% ^B^	17% ^B^	34% ^A^	40% ^C^
1.75–3.00	1% ^B^	3% ^B^	9% ^B^	87% ^A^

Only 2% of the applications in the 4^th^ preliminary score band (1.76 or worse) improved to the 1^st^ final priority score “band” (1.0 to 1.45); and only 1% in the 1^st^ preliminary score “band” (1.00 to 1.45), declined to the 4^th^ final priority score band (1.76 to 3.00). Priority scores of 1.46 to 1.75 are often assumed by many reviewers (often erroneously) as the region of the funding pay line cut-off for ICs. Large changes (0.15 and greater) into or out of the 1.45 to 1.75 range can have substantive practical importance.

## Discussion

There is a moderate correlation (0.78) between the average preliminary and final priority scores for R01 applications reviewed by CSR/NIH. Since the assigned reviewers are also members of the review panel and have been selected for their credibility in peer review and scientific expertise, and that both the panel and assigned reviewers are using a standard set of review criteria, a correlation would be expected.

However, this study also establishes that overall the average of the three preliminary priority scores is significantly different from the final priority score of the SRG (p = <.01). This also holds true when the data set is broken down into experienced applicants, competing renewals (Type 2) or clinical and non-clinical research. The only exception was for the Type 1 New Principal Investigator cohort where the scores were not significantly different.

CSR and NIH have had a history of special commitment to New Principal Investigators [11]. NIH also gives guidance to reviewers to consider the career stage of the applicant at the time of review, particularly as it relates to publication history and preliminary data. One possible explanation for no pre to post discussion difference could be that both assigned reviewers and study section members are taking the career stage of New Principal Investigator applicants into consideration.

Unless the difference between the preliminary and final priority score leads to an adjustment of such magnitude as to affect further IC discussion and decisions, the differences are of little consequence. In this study approximately 13.3% of applications with priority scores of 1.75 or better have final priority scores that differ by 0.15 or more from their average preliminary score. In recent years such changes could be of considerable importance to ICs, and contribute to their discussions on funding.

The NIH has recently announced [12] that they have modified the priority score scale used by reviewers to a single digit, rather than to a decimal. They have also announced a revision of the review criteria and the addition of a new class of applications, the Early Stage Investigators. These modifications were implemented beginning with the October 2009 review round. Evaluation of the impact of these modifications on peer review has just begun.
